# Q-uestioning the Diagnosis: An Educational Case Report

**DOI:** 10.1177/20543581221097749

**Published:** 2022-05-02

**Authors:** Aran Thanamayooran, Karthik Tennankore, Laurette Geldenhuys, Elana Murphy, Keigan More

**Affiliations:** 1 Dalhousie University, Halifax, NS, Canada; 2 Division of Nephrology, Department of Medicine, Dalhousie University, Halifax, NS, Canada; 3 Division of Anatomical Pathology, Department of Pathology, Dalhousie University, Halifax, NS, Canada; 4 Division of Rheumatology, Department of Medicine, Dalhousie University, Halifax, NS, Canada

**Keywords:** Q fever, glomerulonephritis, glomerular diseases, infectious diseases, AKI (acute kidney injury)

## Abstract

**Rationale:**

Q fever is a zoonotic infection that may lead to acute or long-term renal injury. Given its rare incidence, Q fever is not often considered on the initial differential diagnosis for glomerular disease which can lead to delays in treatment. This case highlights the importance of avoiding early diagnostic closure and revisiting the differential diagnosis in the setting of an atypical clinical presentation or response to treatment.

**Presenting Concerns:**

A 52-year-old female was referred for assessment of possible glomerulonephritis. She described a 3-month history of bilateral lower extremity rash, intermittent knee pain with swelling, and a 2-year history of subjective fevers. Urinalysis showed persistent microscopic hematuria, and her creatinine was elevated at 94 umol/L (baseline 59 umol/L). Her initial investigations included an elevated C-reactive protein (CRP) and rheumatoid factor with a weakly positive anti nuclear antibody (ANA).

**Diagnoses:**

Kidney biopsy was consistent with an immune complex mesangial proliferative glomerulonephritis. Light microscopy showed diffuse global mesangial hypercellularity. Immunofluorescence was positive for trace mesangial IgG and kappa, 1+ IgM, lambda and C1q, and 2+ C3. Electron microscopy showed mesangial electron dense deposits. These findings were felt to be most in keeping with mesangial proliferative lupus nephritis; however, it was acknowledged that clinical and laboratory findings supporting this diagnosis were lacking.

**Interventions:**

Following treatment with oral prednisone her symptoms resolved, and renal function improved. However, she was unable to taper off prednisone completely without her symptoms returning. Additional immunosuppressive therapies were trialed, but she remained steroid dependent with disease flares related to prednisone tapers. Her atypical response to treatment led to consideration of alternative diagnoses, and further investigation revealed positive Q fever serology (phase-I IgG 1:1892, phase II IgG 1:8192, phase-I and -II IgM < 1:16). She was diagnosed with long-term Q fever and was treated with doxycycline and hydroxychloroquine.

**Outcomes:**

She remained on treatment for 2 years. During this time, her symptoms resolved, hematuria disappeared, and her creatinine returned to baseline. Following cessation of therapy, her Q fever IgM titres rose, and she was restarted on doxycycline and hydroxychloroquine indefinitely.

**Teaching Points:**

(1) Keeping a broad differential diagnosis in the setting of atypical clinical features or unexpected response to therapy is important for ensuring accurate diagnosis and appropriate treatment. (2) Clinical improvement in relation to immunosuppressive therapy does not preclude an infectious cause of glomerular disease.

## Abrégé

**Justification:**

La fièvre Q est une infection zoonotique qui peut entraîner l’insuffisance rénale aiguë ou chronique. D’incidence rare, la fièvre Q n’est généralement pas considérée dans le diagnostic différentiel initial de la glomérulonéphrite, ce qui peut retarder le traitement. Ce cas souligne l’importance d’éviter de poser un diagnostic précoce et d’envisager un diagnostic différentiel lors d’une présentation clinique atypique ou d’une réponse inattendue au traitement.

**Présentation du cas:**

Une femme de 52 ans envoyée pour l’évaluation d’une possible glomérulonéphrite. La patiente disait avoir une éruption cutanée bilatérale des membres inférieurs et une douleur intermittente au genou avec enflure depuis trois mois, ainsi que des épisodes de fièvre subjective au cours des deux dernières années. L’analyze d’urine a révélé une hématurie microscopique persistante et un taux de créatinine élevé à 94 umol/L (59 umol/L initialement). Ses premiers examens ont montré une élévation de la CRP et du facteur rhumatoïde, ainsi qu’un titer d’ANA faiblement positif.

**Diagnostic:**

La biopsie rénale était compatible avec une glomérulonéphrite mésangiale proliférative à complexe immun. La microscopie optique a démontré une hypercellularité mésangiale diffuse globale. L’immunofluorescence des cellules mésangiales a révélé des traces d’IgG, de kappa, d’IgM, de chaînes lambda et de fragments C1q 1+ et de C3 2+. La microscopie électronique a montré des dépôts denses aux électrons en localization mésangiale. Ces résultats ont été jugés comme plus conformes à la néphrite lupique mésangiale proliférative; on a toutefois reconnu que des résultats cliniques et de laboratoire appuyant ce diagnostic faisaient défaut.

**Intervention:**

Après un traitement par prednisone orale, les symptômes de la patiente se sont résorbés et sa fonction rénale s’est améliorée. Il ne lui a toutefois pas été possible de cesser complètement la prednisone sans une réapparition de ses symptômes. D’autres traitements immunosuppresseurs ont été mis à l’essai, mais la patiente est restée dépendante des stéroïdes en raison de poussées de la maladie liées aux réductions de la dose de prednisone. Cette réponse atypique au traitement a mené l’équipe soignante à envisager d’autres diagnostics. Des examens supplémentaires ont révélé une sérologie positive pour la fièvre Q (IgG phase I 1:1892; IgG phase II 1:8192; IgM phase I et II <1:16). La patiente a reçu un diagnostic de fièvre Q chronique et a été traitée avec de la doxycycline et de l’hydroxychloroquine.

**Résultats:**

La patiente a reçu le traitement pendant deux ans au cours desquels ses symptômes se sont résorbés, son taux de créatinine est revenu à la valeur initiale et l’hématurie est disparue. Après l’arrêt du traitement, ses titres d’IgM de fièvre Q ont augmenté et la patiente a dû reprendre indéfiniment le traitement par doxycycline et hydroxychloroquine.

**Enseignements tirés:**

(1) Pour garantir un diagnostic précis et le traitement approprié, il est important d’envisager un diagnostic différentiel étendu en présence de caractéristiques cliniques atypiques ou d’une réponse inattendue au traitement. (2) L’amélioration clinique liée au traitement immunosuppresseur n’exclut pas une étiologie infectieuse de la glomérulonéphrite.

## Introduction

Q fever is a zoonotic illness caused by the gram-negative bacteria *Coxiella burnetti*. Typical bacteria reservoirs include sheep, cattle, and goats although infection from exposure to other animals have been reported.^1^ Human infection typically occurs following inhalation of aerosolized bacteria and can lead to either acute or long-term illness. Clinical presentations are highly variable and depend on the strain of *C. burnetti* in addition to host factors. Signs and symptoms of acute Q fever infection tend to develop following a 2- to 3-week incubation period and may include flu-like illness, pneumonia, and/or hepatitis. Less common, but potentially life-threatening, presentations of acute Q fever including meningitis and pericarditis have also been reported.^1^ Long-term Q fever is often associated with hematologic spread and may manifest clinically as endocarditis, osteomyelitis, and/or endovascular graft infection.^2^ While renal dysfunction is not a classical Q fever presentation, case reports have described an association between glomerulonephritis and long-term *C. burnetti* infection.^3^ Herein, we describe a case of glomerulonephritis ultimately attributed to an occult long-term Q fever infection.

## Presenting Concerns

A 52-year-old female with microscopic hematuria and a creatinine of 94 umol/L (baseline 59 umol/L) was referred to nephrology for the assessment of possible glomerulonephritis. She described a 3-month history of bilateral lower extremity rash, intermittent right knee pain with swelling, and a 2-year history of subjective fevers and sweats. She had been previously treated with a short course of oral prednisone by her family physician with effect, but her rash and symptoms returned soon after cessation of treatment. She was seen by rheumatology the week prior to her nephrology consultation and was restarted on prednisone 30 mg daily due to concern over possible connective tissue disease and/or vasculitis.

## Clinical Findings

Her past medical history was significant for microscopic hematuria of at least 6 months duration with normal cystoscopy, left femoral crossover graft in 2005 due to right iliac artery disease, gastroesophageal reflux, dyslipidemia, and depression. Her medications included rabeprazole, acetylsalicylic acid (ASA), simvastatin, citalopram, and bupropion.

Examination revealed a palpable petechial rash in her lower extremities and moderate effusions in her right knee and ankle which were warm to touch. The remainder of her examination, including vital signs, was within normal limits.

Initial laboratory investigations revealed a normocytic anemia (hemoglobin 102 g/L), and mild leukocytosis (white blood cells [WBC] 11.12 × 10^9^/L). Her creatinine was elevated at 94 umol/L compared to a baseline of 59 umol/L earlier in the year. Urinalysis revealed trace proteinuria, and urine microscopy showed 58 red blood cells/HPF, acanthocytes, and 7 WBC/HPF. Rheumatic investigations showed an elevated erythrocyte sedimentation rate 115 mm/hr (normal 0-20), C-reactive protein 94 mg/L (normal 0-8), and rheumatoid factor 176 IU/mL (normal 0-20). Antineutrophil cytoplasmic antibodies, C3, and C4 were within normal limits. A previously arranged biopsy of her petechial rash was consistent with a leukocytoclastic vasculitis and immunofluorescence (IF) was positive for dermal capillary IgM and C3. Arthrocentesis of her knee was not consistent with a crystalline or infectious arthropathy.

## Diagnostic Focus and Assessment

Additional investigation into the cause of her kidney dysfunction included negative hepatitis B, hepatitis C, HIV, and syphilis serology as well as negative blood cultures. Serum protein electrophoresis did not show a monoclone, cryoglobulins were negative and anti-CCP was normal. Kappa and lambda light chains were minimally elevated at 15.2 g/L (normal 6.47-12.71) and 12.8 g/L (normal 2.43-5.91), respectively. Her antinuclear antibody test was weakly positive with RNP 1.1 AI (normal < 1.0 AI). Her dsDNA was negative at 2 IU/mL (normal ≤ 4 IU/mL) and her lupus anticoagulant ratio was normal at 1 (normal 0.8-1.2). She had 0.32 g of protein on a 24-hour urine collection and her renal ultrasound showed normal-sized kidneys without increased echogenicity.

**Figure 1.**
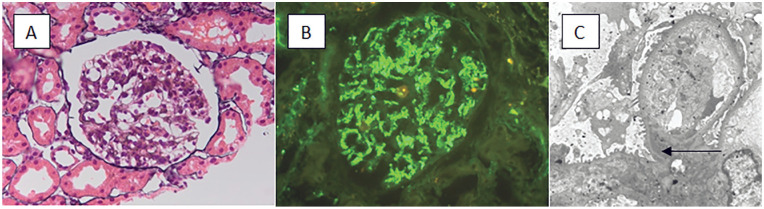
(A) Light microscopy (Jones Silver Stain 400×) showing mesangial hypercellularity. (B) Immunofluorescence for immunoglobulin M (IgM) (1+ intensity, 400×). (C) Transmission electron microscopy with electron dense deposits (arrow) and mesangial hypercellularity (4,000×).

Renal biopsy was not initially pursued given concern over a low diagnostic yield as her symptoms and creatinine improved significantly while on prednisone therapy. However, after stopping prednisone, her creatinine rose to 111 umol/L and her symptoms returned which prompted a renal biopsy. Renal pathology was in keeping with an immune complex mesangial proliferative glomerulonephritis. Diffuse global mesangial hypercellularity was seen on light microscopy and IF showed trace mesangial IgG and kappa, 1+ IgM, lambda and C1q, and 2+ C3. Electron microscopy showed mesangial electron dense deposits (Figure 1). Her pathology findings were felt to potentially be in keeping with mesangial proliferative lupus nephritis; however, it was acknowledged that clinical and laboratory findings supporting this diagnosis were lacking.

## Therapeutic Focus and Assessment

Following renal biopsy, and in the absence of an alternative diagnosis, she was restarted on prednisone 30 mg daily for suspected lupus and her creatinine improved to 75 umol/L. Azathioprine was started as a steroid sparing agent, and her prednisone was weaned off. Unfortunately, she was unable to tolerate azathioprine due to gastrointestinal side effects and her creatinine increased and clinical symptoms returned following completion of her steroid wean. She was subsequently started on mycophenolate mofetil (MMF) with a target dose of 1 g twice daily in addition to prednisone 10 mg daily under the guidance of rheumatology. On this regimen, her creatinine improved to 73 umol/L but once again increased to 109 umol/L when steroids were stopped despite continuing with MMF. In response, her prednisone was restarted, and hydroxychloroquine was initiated at 200 mg twice daily. A dose of rituximab was arranged through rheumatology followed by a prednisone taper but unfortunately her symptoms recurred yet again.

At this time her lupus diagnosis was reconsidered given atypical clinical features and her inability to wean off prednisone. Further infectious work up, including transthoracic echocardiogram, computed tomography (CT) scan of the abdomen and pelvis, and repeat blood cultures did not reveal an infectious source. She was referred to an infectious disease consultant who ordered additional investigations including Q fever serology which ultimately returned positive (phase-I IgG 1:1892, phase-II IgG 1:8192, phase-I and -II IgM <1:16). Despite living in a rural setting, no direct zoonotic exposure was identified. Based on the pattern of phase-I and -II antibody titres in conjunction with the patient’s clinical course, the infectious disease team felt this presentation was most in keeping with long-term Q fever and initiated treatment consisting of doxycycline 100 mg BID and hydroxychloroquine 200 mg TID. Simultaneously, her MMF was stopped, and her prednisone was weaned off. Early in her Q fever treatment course, she underwent a revision of her femoral crossover graft due to occlusive thromboembolism. Intraoperative cultures were negative, but her Q fever treatment duration was extended to 2 years as the infectious disease team felt her vascular thromboembolism was likely in keeping with endovascular Q fever infection.

## Follow-Up and Outcomes

**Figure 2.**
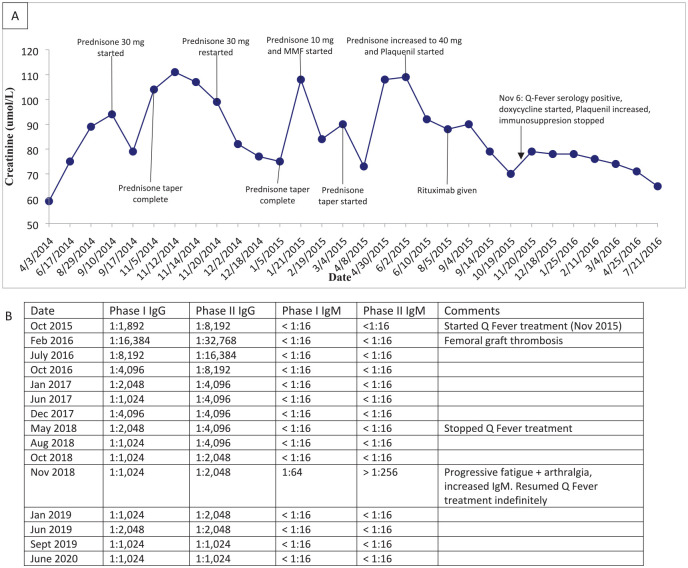
Renal function (A) and Q fever serology (B) response to treatment. Note. IgG = Immunoglobulin G; IgM = Immunoglobulin M.

While being treated for Q fever, her creatinine nadired at 60 to 70 umol/L, her hematuria and proteinuria disappeared, and her subjective fevers and chills resolved in conjunction with falling Q-fever titres (Figure 2). Hydroxychloroquine and doxycycline were discontinued after 2 years but were restarted shortly thereafter due to a rising Q-fever IgM titer with a plan for likely lifelong therapy.

## Discussion

Long-term Q fever is an uncommon zoonotic infection which has been associated with glomerular injury in the literature (Table 1.). Here we describe a case of immune complex mesangial proliferative glomerulonephritis initially felt to be related to class-II lupus nephritis but later determined to be a result of long-term Q fever, potentially from an endovascular source. This case highlights the difficulty in establishing the diagnosis of Q fever as a result of non-specific symptoms and a high degree of variability in clinical presentation. In addition, Q fever may be overlooked when infectious causes are considered as *C. burnetti* does not stain with the traditional gram technique^1^ and requires specific blood culture media to grow.^4^

**Table 1.** Long-term Q Fever Associated With Biopsy Proven Glomerulonephritis.

Case	Age/sex	Light microscopy	Immunofluorescence	Electron microscopy	Renal diagnosis	Q fever treatment	Renal outcome
1. Marmion et al^5^	48M	Not available	Not available	Not available	Long-term lobular glomerulonephritis	None	Death
2. Ferguson et al^6^	48M	Basement membrane thickening	Not available	Not available	Membranous glomerulonephritis	Tetracycline	Death
3. Dathan and Heyworth^7^	58M	Mesangial hypercellularity and endothelial proliferation	Granular IgG, IgA, and C3	Mesangial deposits, foot process fusion	Immune complex glomerulonephritis	Cotrimoxazole	Death
4. Uff and Evans^8^	39F	Mesangial proliferation, capillary loop thickening, and mesangial interposition	IgM and C3	Not available	Diffuse lobular glomerulonephritis	Tetracycline and lincomycin	Death
5. Rosman et al^9^	28F	Mesangial hypercellularity	Granular IgM and C3	Subendothelial deposits, podocyte fusion	Immune complex nephritis	Lincomycin and tetracycline	Improved
6. Perez-Fontan et al^10^	33M	Mesangial expansion and hypercellularity	Granular mesangial IgM and C3	Not available	Focal and segmental proliferative glomerulonephritis	Doxycycline and lincomycin	Improved
7. Perez-Fontan et al^10^	38M	Fibrotic crescents, proliferative intracapillary changes, and segmental necrosis	Granular mesangial IgM and C3	Not available	Focal and segmental proliferative glomerulonephritis	Doxycycline	Death
8. Perez-Fontan et al^10^	37F	Mesangial expansion and hypercellularity	Granular mesangial IgM and C3 with weak C4 and C1q	Not available	Diffuse proliferative glomerulonephritis	Doxycycline and cotrimoxazole	Resolved
9. Gerlis et al^11^	27M	Focal and segmental proliferation	Not available	Not available	Focal and segmental proliferative glomerulonephritis with crescents	None	Death
10. Vacher-Coponat et al^12^	69M	Segmental mesangial proliferation and double contours of basement membrane	Diffuse IgG, IgM, C3, and C1q	Not Available	Focal and segmental proliferative glomerulonephritis	Doxycycline and chloroquine	Resolved
11. Leclerc et al^3^	64M	Global mesangial and endocapillary hypercellularity	C3, IgG, IgA, C1q, kappa, lambda, and IgM(1+) in capillary walls	Subendothelial deposits and foot process effacement	Immune complex mediated membranoproliferative glomerulonephritis	Doxycycline, hydroxychloroquine, prednisone and MMF	Resolved

*Note*. MMF = mycophenolate mofetil; IgG = Immunoglobulin G; IgA =Immunoglobulin A; IgM =Immunoglobulin M.

The association between Q fever infection and glomerular injury has been previously described (Table 1). We identified 11 cases of long-term Q fever associated with biopsy-proven glomerular injury in the literature over a 60-year time period. Outcomes for these patients were variable. Six patients died, 3 had resolution of renal impairment, and 2 had partial improvement in renal function. The mainstay of treatment was tetracycline class antibiotics (69% of cases), with additional treatments including lincomycin and immunosuppression. Renal biopsy comparison was limited by variation in biopsy technique, analysis, and reporting, given the long period of time between the first and last published cases. Previously published light microscopy findings typically showed mesangial hypercellularity and/or proliferation which is similar to the light microscopy results in our case. Eight cases included IF findings. IgM was found in 88% of these cases, often in association with C3. Our IF findings were consistent with those previously published and demonstrated both IgM and C3 in addition to IgG, C1q, kappa, and lambda. A full-house pattern was not seen but has been reported previously in a patient with long-term Q fever infection.^3^ Only 3 previously published cases described electron microscopy findings. Foot process effacement/fusion was seen in all 3 cases whereas subendothelial electron dense deposits were seen in 2 cases and mesangial electron dense deposits were seen in the remaining case. Our electron microscopy findings included electron dense mesangial deposits; however, unlike prior cases foot process effacement was not noted.

This case highlights 2 important teaching points. First, it stresses the importance of formulating a broad differential, especially in light of an atypical clinical presentation, which may be revisited in the event of an uncharacteristic response to treatment. In our case, a presumptive diagnosis of mesangial proliferative lupus nephritis was made after renal biopsy as the initial work-up failed to identify a clear alternative cause. However, despite appropriate initial therapy, our patient did not improve as expected, prompting a re-evaluation of the case, which ultimately led to the correct diagnosis and management strategy.

The second teaching point is that glomerular injury secondary to infectious causes may respond, at least in part, to treatment with immunosuppressive therapies. In our case, the patient’s early improvement with immunosuppression was felt to support the underlying diagnosis of mesangial proliferative lupus nephritis and led clinicians away from an alternative infectious cause as it may seem counterintuitive for an infectious process to improve with immunosuppression. In this case, it is likely that the repeated improvement with prednisone represented suppression of the immunologic response to a long-term infective process, leading to diagnostic confusion.

Challenging medical cases have a tendency to evolve over time as new information becomes available. Stepping back to reconsider a complex case using information not available at its onset can help identify inconsistencies with prior diagnostic reasoning which may lead to an alternative diagnosis. In our case, this led to the reconsideration of an infectious cause, a diagnosis of long-term Q fever, and appropriate treatment with normalization of renal function.
